# Bovine Clinical *E. coli* Mastitis in Italian Dairy Herds Is Not Associated with a Specific Pathotype

**DOI:** 10.3390/pathogens14111181

**Published:** 2025-11-18

**Authors:** Giulia Laterza, Gabriele Meroni, Alessio Soggiu, Piera Anna Martino, Valerio Massimo Sora, Francesca Zaghen, Luigi Bonizzi, Luciana Colombo, Alfonso Zecconi

**Affiliations:** 1 Department of Biomedical, Surgical and Dental Sciences-One Health Unit, School of Medicine, University of Milan, Via Pascal 36, 20133 Milan, Italy; giulia.laterza@unimi.it (G.L.); alessio.soggiu@unimi.it (A.S.); piera.martino@unimi.it (P.A.M.); valerio.sora@unimi.it (V.M.S.); luigi.bonizzi@unimi.it (L.B.); alfonso.zecconi@unimi.it (A.Z.); 2 Department of Clinical and Community Sciences, School of Medicine, University of Milan, Via Celoria 22, 20133 Milan, Italy; 3 Associazione Regionale Allevatori della Lombardia, Via Kennedy 30, 26013 Crema, Italy; l.colombo@aral.lom.it

**Keywords:** *E. coli*, mastitis, pathogenesis, genomic analysis

## Abstract

Background: *Escherichia coli* is a cause of severe clinical bovine mastitis; however, it is not yet fully understood what makes mastitis-associated bacteria different from commensal strains at the genetic level. The goal of this study was to compare the genomic features, sequence types, virulence, and antibiotic resistance profiles of *E. coli* isolated from healthy cows and cows with clinical mastitis in Northern Italy. Methods: Between 2023 and 2024, 46 *E. coli* isolates, 23 from healthy animals and 23 from mastitis cases were recovered. Standard phenotypic approaches and Oxford Nanopore sequencing were used to investigate the genomic landscape of the strains. Results: Phylogroups A and B1 were the most common in both groups. MLST showed several types, with ST10 (19.6%), ST58 (13.0%), and ST69 (8.7%) being the most common. There was no lineage that was uniquely able to describe the isolates as Mammary Pathogenic *Escherichia coli* (MPEC); indeed, the ST distribution and phylogeny were the same in both groups. A total of 47.8% of isolates had antimicrobial resistance determinants, with β-lactamases (21.7%) and *tetA* (15.2%) being the most common. No significant differences in resistance rates were observed between mastitis and healthy isolates. Pangenome investigation found a large pool of accessory genes, but no genomic signature that distinguished mastitis from commensal isolates across the MPEC. Conclusions: Bovine *E. coli* isolated from milk of both healthy and mastitic cows share sequence types, resistance rates, and accessory genome content, supporting the absence of a unique MPEC pathotype and highlighting the ecological versatility of these bacteria.

## 1. Introduction

Bovine mastitis remains a significant health issue in the dairy industry, resulting in considerable economic losses and impacting animal health and milk quality. *Escherichia coli* (*E. coli*) is one of the primary pathogens implicated in acute mastitis [1].

*E. coli* can be classified into six major phylogenetic groups: A, B1, B2, D, F, and E [2]. Pathogenic *E. coli* strains are usually categorized into several pathotypes depending on their virulence characteristics, disease-causing ability and localization of the infection (e.g., Enterotoxigenic *E. coli* (ETEC), mainly found in human diseases in developing countries; Enteropathogenic *E. coli* (EPEC), linked to diarrhea in infants; and Shiga toxin-producing *E. coli* (STEC), responsible for causing severe foodborne disease) [2]. *E. coli* involved in mastitis differs from the classical pathogenic groups. Indeed, the pathogenesis of *E. coli* mastitis is based on the release of LPS from the bacteria, and not to the presence of specific genes [3,4]. Recent papers suggest a different hypothesis that involves the presence of specific genetic characteristics beyond the development of *E. coli* mastitis implying the possibility to classify them as Mammary Pathogenic *E. coli* (MPEC) [5,6,7]. However, the specific characteristics of MPEC have not yet been described despite extensive research over the last few years [8]. Strains associated with this suggested pathotype seem to exhibit increased proliferation in milk and are more resistant to phagocytosis. However, they lack the most common virulence factors that are usually linked to pathogenicity in other types of infections [3,9]. On the other hand, this description does not adequately explain numerous features of MPEC. For instance, mastitis isolates are substantially less genetically varied than bovine commensal isolates [8], and not all *E. coli* may induce clinical mastitis in experimental models of the disease [10]. As a result, the complete etiology of mastitis caused by *E. coli* is not fully understood.

To contribute in reducing the knowledge gap on this topic, we performed a comparative genomic analysis of 46 *E. coli* strains isolated from quarter milk samples of Italian dairy cows, healthy or with clinical mastitis, to ascertain the genomic differences in terms of chromosome dimension, specific virulence factors and antibiotic resistance genes, as well as determination of sequence type (ST) to corroborate scientific knowledge about MPEC.

## 2. Materials and Methods

### 2.1. Bacterial Isolation and Identification

Bovine *E. coli* isolates were obtained from quarter milk samples taken in dairy herds of Lombardy region and isolated in the laboratory of the Associazione Regionale Allevatori della Lombardia (ARAL, Crema, Italy) between 2023 and 2024. Twenty-three *E. coli* from healthy cows and 23 *E. coli* associated with mastitis were included in this study. Quarter milk samples (QMS) were collected aseptically from bovine udders, following the National Mastitis Council (NMC) guidelines to minimize contamination. Samples were immediately refrigerated at 4 °C and processed within 24 h of collection in ARAL laboratories. The isolates from healthy cows were selected from routine post-calving QMS showing the presence of a pure culture of suspected *E. coli*. Clinical isolates were collected from QMS sent to the ARAL lab for diagnosis of mild/severe mastitis. Only one isolate/herd for each category were collected; therefore, the isolates came from 38 different herds. For isolation, 10 µL of each milk sample was streaked onto MacConkey agar plates and incubated aerobically at 37 °C for 18–24 h. Lactose-fermenting, typically pink and medium to large colonies, were subcultured onto eosin methylene blue (EMB, Microbiol, Sardinia, Italy) agar to confirm the presence of *E. coli*. Colonies exhibiting a characteristic metallic green sheen on EMB were considered presumptive *E. coli*. These colonies were further subjected to Gram staining, revealing Gram-negative rods, and confirmed by Vitek™ system (Biomerieux, Lion, France). After isolation and identification, bacteria were streaked onto blood agar plates and then delivered to the laboratory of the One Health unit of the Department of Biomedical, Surgical and Dental Sciences in Milan, where a single colony from each sample was resuspended in Tryptic Soy Broth (TSB, Microbiol, Sardinia, Italy) and incubated overnight at 37 °C. The next day, 50 µL of bacterial cultures were spread on Tryptic Soy Agar (TSA) and incubated at 37 °C for 24 h.

### 2.2. DNA Extraction, and Nanopore Sequencing Library Preparation

Isolated colonies on TSA were resuspended straight in 750 µL of the lysis solution from ZYmoBIOMICS™ DNA Miniprep Kit (Zymo Research, Irvine, CA, USA), and DNA was extracted following manufacturer’s instructions. DNA quality and quantity was assessed using NanoReady Touch series Micro Volume (UV-Vis) spectrophotometer (Aurogene, Rome, Italy), making sure that the A_260_/A_280_ and A_260_/A_230_ ratios fell between 1.8 and 2, respectively.

The Rapid Sequencing DNA V14-barcoding kit (SQK-RBK114.96; Oxford Nanopore Technologies, ONT, Oxford, UK) was used to barcode each sample, which included 200 ng of DNA. Twelve barcoded samples were loaded in MinION FLO-MIN114 v10.4.1 flow cell (ONT, Oxford, UK) and sequenced in a MinION Mk1C (ONT, Oxford, UK) for maximum 72 h.

### 2.3. Bioinformatic Genome Sequencing Analysis

The same workflow analysis described elsewhere was used [11]. Briefly, .pod5 files were basecalled (--dna_r10.4.1_e8.2_400bps_hac@v5.0.0), adapters trimmed, and demultiplexed (--config configuration.cfg --barcode_kits SQK-RBK114.96 --trim_barcodes; min_score threshold default 60) with Dorado (v0.8.2) on GPU A100 HPC. Summary statistics were obtained with NanoPlot (v1.44.0) (--verbose --tsv_stats --N50 --fastq). Reference guided filtration with a 1 kbp threshold was achieved using FiltLong (v0.2.1), blasting mastitis-associated (MA) *Escherichia coli* against a reference strain AJQW01000001.1 (referred to as ‘Ref_xx.fasta’) (--assembly Ref_xx.fasta --trim --min_lenght 1000 --keep_percent 90). Healthy-derived genomes were de novo assembled using Flye (v2.8.1-b1676) (--nano-corr --genome-size 5 m --asm-coverage 50 --trestle). Assembled contigs were polished with Medaka (v2.0.1) (medaka_consensus --t 12 --m dna_r10.4.1_e8.2_400bps_hac@v4.1.0). Genomic completeness and contamination were derived using CheckM2 (v.1.0.2) (check2m predict --threads 12 --x fna). The NCBI Prokaryotic Genome Annotation Pipeline (PGAP) was used to annotate genomes and find out the total numbers of coding sequences, rRNAs, and tRNAs. Multilocus sequence types (MLSTs) were determined by uploading the genomes to the Center for Genomic Epidemiology (https://www.genomicepidemiology.org/; accessed on 28 October 2024), using the online tool for MLST prediction. Finally, pan-genomes were visualized using both an online tool (IPGA) and a command-line pipeline (ANVI’O).

### 2.4. Bioinformatic Analysis of Antimicrobial-Resistance Genes and Virulence Factors

To determine whether antibiotic-resistance genes (ARGs) were present, the Comprehensive Antibiotic Resistance Database (CARD), the National Centre for Biotechnology Information (NCBI), ResFinder, Plasmidfinder, and the Virulence Factor, were all consulted for genomes analysis using Abricate (v1.0.1) (abricate/path/to/fna/*.fna --db card, vfdb, resfinder, ncbi, plasmidfinder –minid 95 –csv > /path/to/output/.csv). A threshold identity of ≥95% was established for each sample in the study.

### 2.5. Statistical Analysis

In this study customized bioinformatic pipelines were used to analyse genomic data. All categorical data were analyzed using χ^2^ Test. Quantitative data were analyzed using a one-way ANOVA test and Dunnett’s multiple comparisons test in GraphPad Prism 10; the quantitative data were presented as the mean and standard deviation (SD).

## 3. Results

### 3.1. General Features of the Genomes

After genome assembly, the summary statistics of the sequencing data from a Na-nopore Mk1C include a mean coverage of 184.6× with a N50 of 3 731 408,13 bp, a genome size of 4,962,502.283 bp, and a GC% content of 51. The whole-genome assemblies were deposited at GenBank repository under the bioproject PRJNA1242576. Table 1 summarizes the main statistics of the polished and assembled genomes.

This study analyzed 46 *E. coli* isolates from *Bos taurus* milk in Italy, consisting of 23 isolates from healthy animals (H group) and 23 from animals with clinical mastitis (MA group), and characterize genomic features in relation to bovine health status. The assembly level exhibited variability, with the number of contigs per assembly ranging from a single unit (noted in five isolates, specifically H_EC26, H_EC43, H_EC77, MA_EC101, and MA_EC102) to a maximum of 45 (H_EC55). The median fragment count was 4, with a mean of 6.8 fragments (SD = 8). No significant changes in fragment distribution were seen between the H and MA groups (H median: 3, MA median: 4), suggesting same assembly capability. Complete, circular assemblies (“Y” in the table) were obtained in 11 genomes (24%), distributed across both groups (6 H, 5 MA). The assembled genome sizes were conformed to species expectations, ranging from 4.26 Mbp (MA_EC96) to 5.41 Mbp (MA_EC93), with averages (±SD) of 4.90 ± 0.19 Mbp for H isolates and 4.99 ± 0.21 Mbp for MA isolates. Mastitis-associated isolates exhibited many high-mass outliers (≥5.3 Mbp genome size: MA_EC89, MA_EC93), indicative of augmented accessory gene richness, despite minor overall group differences (mean difference: +0.09 Mbp favouring MA). GC content exhibited notable stability across all isolates, averaging 51% (mean: 51.02%, SD: 0.19%), with H_EC55 being slightly elevated at 52%. Gene annotation estimated a total coding sequence (CDS) count ranging from 4257 (MA_EC96) to 5192 (MA_EC89). The average CDS count for healthy isolates was 4659 (SD: 142), whereas the MA group showed an average of 4684 CDS (SD = 175), indicating a minor, non-significant increase in the MA group. The sequencing coverage across was predominantly high (mean: 174×, SD: 85×; median: 167×), peaking at 394× in H_EC86 and 396× in MA_EC96, hence guaranteeing base-call precision and dependable identification of genetic variations. Genome completeness, evaluated by CheckM2, consistently reached 100% across all assemblies, providing robust evidence for the absence of substantial assembly gaps or omitted essential genes. The levels of suspected contaminant DNA were consistently low (mean: 0.26%, SD: 0.27%), varying from undetectable (H_EC81: 0.00%) to a peak of 1.29% (H_EC55); just three isolates (all from the H group) surpassed 1% contamination, indicating minimal impact from environmental or laboratory artefacts. Neither completeness nor contamination scores shown variation among health groups.

**Figure 1:**
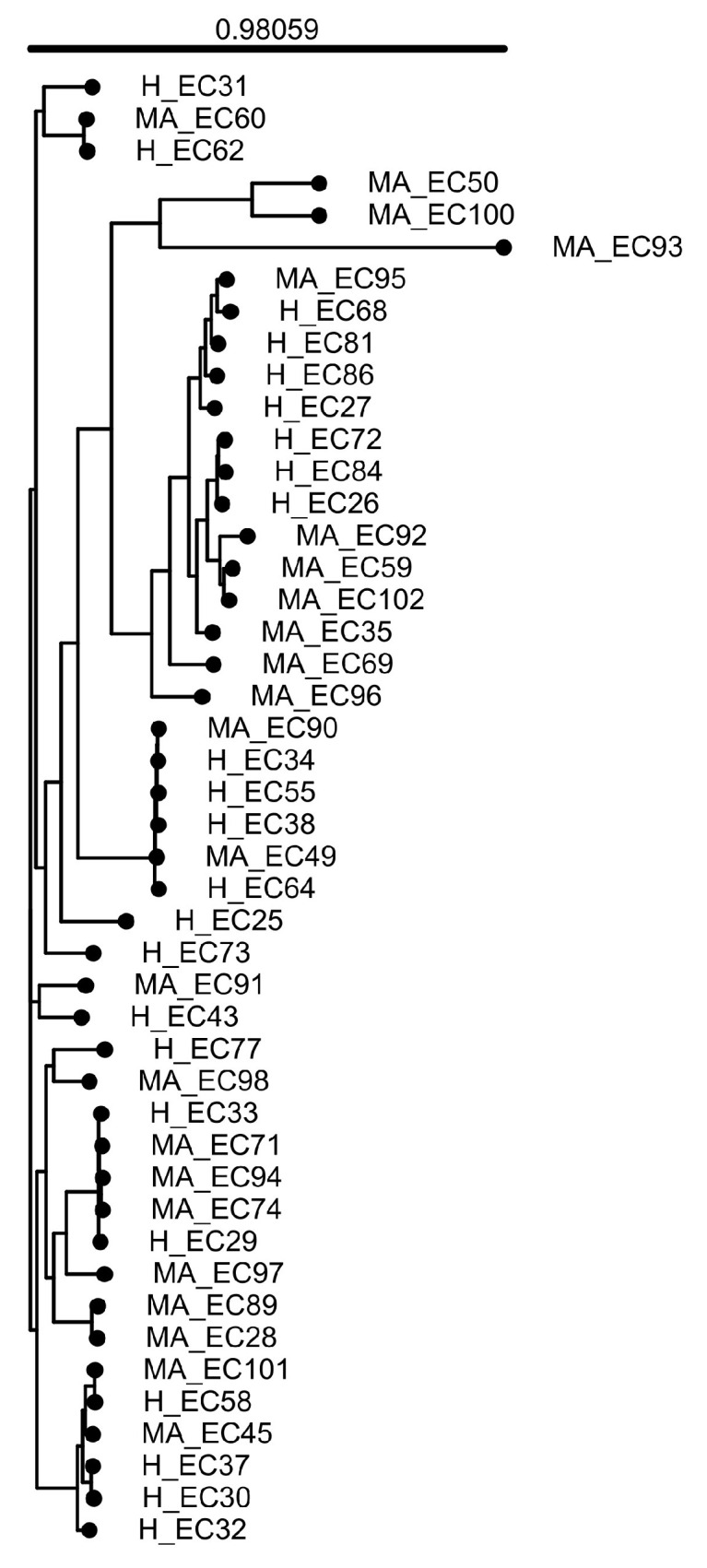
IPGA construction of a whole-genome-based phylogenetic tree. The tree illustrates the evolutionary relationships among *E. coli* strains in this study. The branch lengths reflect genomic differences, while the clustering suggests genetic similarity and possible shared origins. The major branching point, or the evolutionary split from which all strains in the phylogenetic tree originate, has significant statistical support (confidence value, 0.98059). H, healthy; MA, mastitis-associated.

A whole-genome based phylogenetic tree (Figure 1) was built with the online tool Integrated Prokaryotes Genome and pan-genome Analysis service IPGA (v.1.09, https://nmdc.cn/ipga/) (accessed on 10 April 2025).

**Figure 2:**
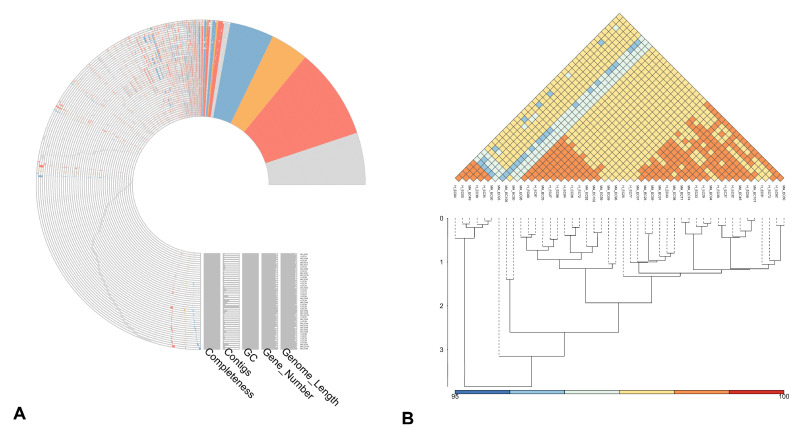
Pan-genome analysis of *E. coli* isolates. (**A**) Pan-genome profile based on gene clusters. (**B**) Heatmap and hierarchical clustering based on pairwise average nucleotide identity (ANI). Colors indicate the degree of average nucleotide identity between each pair, with red corresponding to higher ANI values (more genomic similarity). The hierarchical clustering beneath shows how genomes are grouped together in hierarchical clusters.

**Figure 3:**
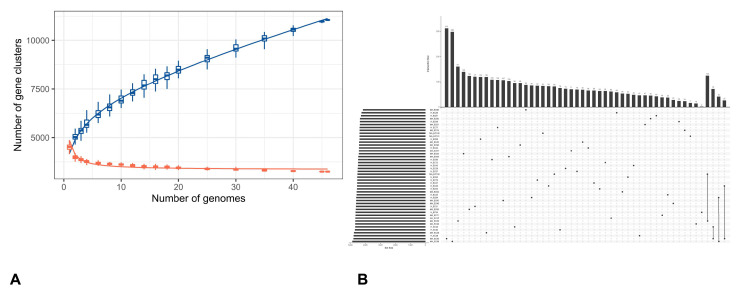
Pan-genome analysis of *E. coli* isolates (Continued): (**A**) Number of pan-gene clusters (blue) and sequenced genomes (orange) among isolates. This type of graph helps to gain information on the genetic variability and evolution of the species under study based on the blue curve shape according to the number of genomes analyzed. More specifically, we can define whether the pan-genome is ‘open’—as in our case—when the number of clusters keeps increasing with the insertion of new sequenced genomes, or ‘closed’, when the growth of the blue line stabilizes. (**B**) Upset plot of comparisons among unique clusters of genes.

Figure 2A reports the pan-genome profile of all *E. coli* strains derived from Clusters of Orthologous Genes (COG) annotation, where the colored components represent the shared core cluster genes by isolates under analysis. More in dept, red indicates genes involved in metabolism (2659), orange represents genes related to information processing and storage (1168), and blue shows genes involved in cellular processes and signaling (1544). Finally, portions colored in grey and left blank pin out poorly characterized (570) or not annotated (5494) genes, respectively. Moreover, the average nucleotide identity (ANI) analysis (Figure 2B) among all strains revealed a few strains with the lowest identity of 96%, while the most show an identity of 98%. On a total of 46 analyzed genomes, 11,046 pan-gene clusters were identified (Figure 3A), with a range from 2 to 313 of the distinct gene clusters in each genome (Figure 3B).

**Figure 4:**
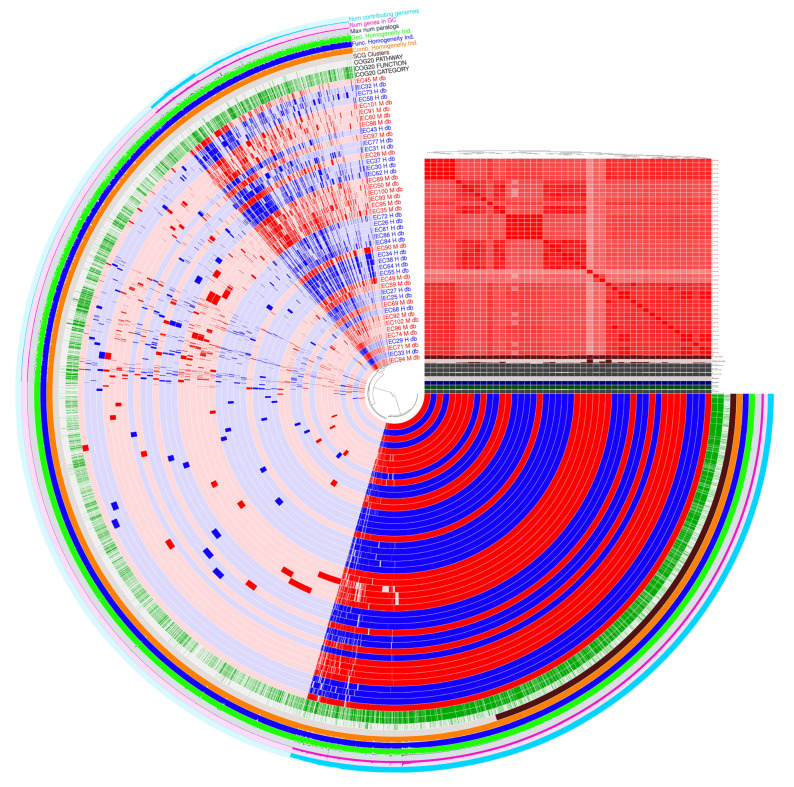
Pangenome visualization of *E. coli* strains visualized by ANVI’ O. Blue and red genomes represent strains derived from healthy donors and associated with mastitis, respectively. Gene cluster presence or absence determines how the samples are grouped in the central dendrogram. The samples are shown in the phylogenetic tree in the order determined by the ANI % of identity. A red square in the evolutionary tree represents each sample cluster, signifying ANI percentage identity values more than 99%.

The pangenome representation derived from ANVI’O (Figure 4), describes more in-depth how our genomes are clustered, reporting how many genes are present compared to the proportion of genomes that include them. The pangenome is composed of different categories: (a) 2882 core genes (99% ≤ strains ≤ 100%), (b) 455 soft-core genes (95% ≤ strains < 99%), (c) 2213 shell genes (15% ≤strains < 95%), and (d) 10,326 cloud genes (0% ≤ strains < 15%).

### 3.2. Pangenome Visualization with PPANGGOLIN Pipeline

The rarefaction curve (Supplementary Figure S1) measures the pangenome diversity across all examined genomes, categorizing gene content into pangenome, soft accessory, soft core, exact accessory, and exact core. The pangenome expands from 5312 to 10,000 gene families across 46 genomes, with no saturation seen in rarefaction analysis according to Heaps’ law. The soft accessory and soft core gene families increase with more genomes, but the soft core stabilizes earlier, marking near-universal genes. The strict core remains constant at about 3196 gene families shared by all strains, likely representing essential functions. Individual genomes contain roughly 4500–4800 genes and 4320–4850 families. MA_EC28 has 4873 genes in 4712 families, including 3572 soft core and 3822 persistent families, while cloud genes range widely from 134 to 778 per genome.

**Figure 5:**
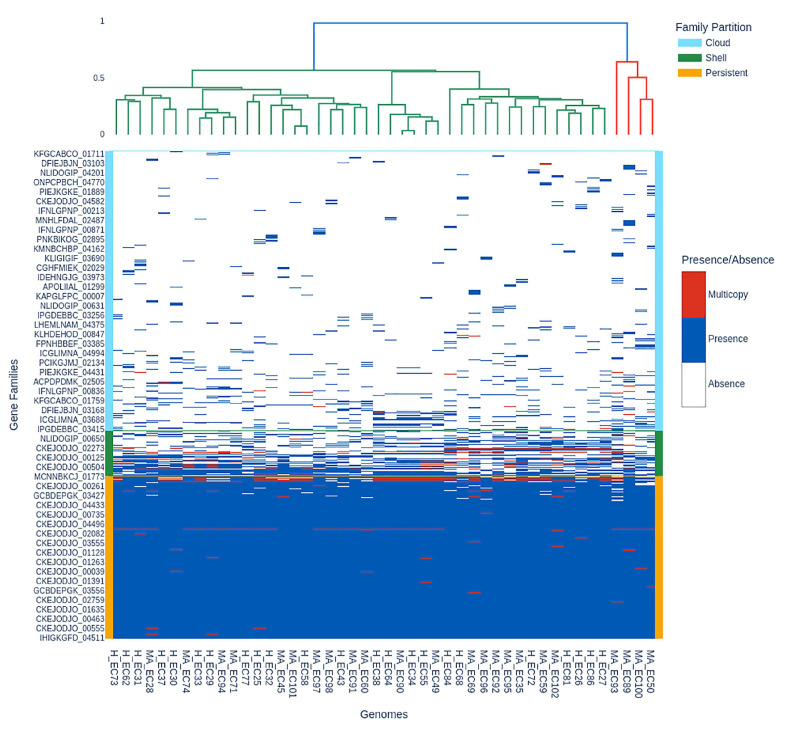
Presence–absence matrix and hierarchical clustering of gene families across H and MA *E. coli* genomes. Persistent (core) families dominate the lower block, variable accessory families partition the upper section, and shell/cloud diversity informs strain clustering.

The presence–absence matrix (Figure 5) lists each gene family (rows) across all included genomes (columns), with colors indicating presence (blue), multicopy (red), and absence (white) and family partition identity (cloud, shell, persistent) on the right and in the dendrogram.

In the lowest half of Figure 5, the blue block represents the persistent genome (median per genome: ~3739–3849 gene families). This matches the rarefaction plateau for persistent and exact core genomes (3196 gene families). The shell and cloud genomes have presence and absence polymorphism, shown by blue and white in the upper matrix. The cloud partition of MA_EC93 has 778 gene families, while H_EC84 has 380. Red indicates multicopy families, including up to 221 families (MA_EC89) in pathogenic isolates, indicating transposons, duplications, or plasmid dynamics. The dendrogram shows that genomes cluster by gene content, matching partition identity.

*E. coli* pangenome evolution is shown by the U-shape gene family frequency distribution (Supplementary Figure S2).

### 3.3. Antibiotic Resistance

**Figure 6:**
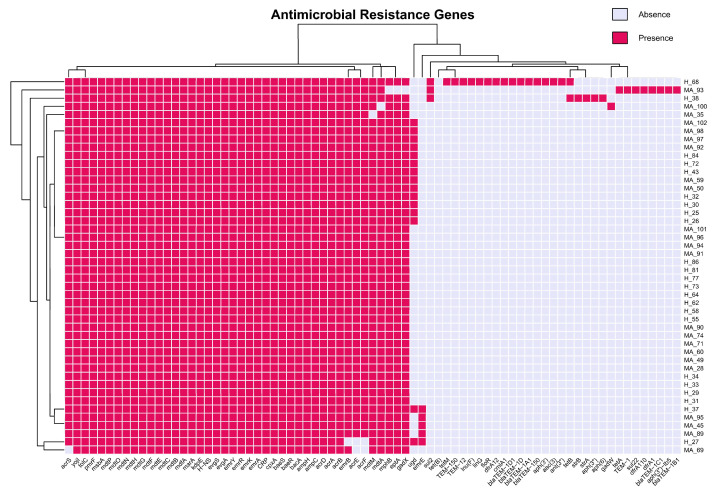
Heatmap of antimicrobial resistance genes in *E. coli*. Fuchsia, Presence; Liliac, Absence.

In Figure 6 we detected a 100% prevalence (46/46) of efflux pump-related ARGs for multidrug-resistant Gram-negative bacteria as well as resistance to beta-lactam antibiotics (*ampC*, *ampH*). In addition, *bacA*, a resistance gene that alters or deactivates the bacitracin and *pmrF*, a colistin-resistance gene that acts on changes at the level of the lipopolysaccharide of the exterior wall of Gram-negative bacteria, are found in all samples. There are no significant statistical differences between healthy and mastitis-associate groups (χ^2^ Test). Moreover, it is showable that most shared resistance genes are present in all our samples, even though some bacteria present specificity, but not necessarily to create clusters.

**Figure 7:**
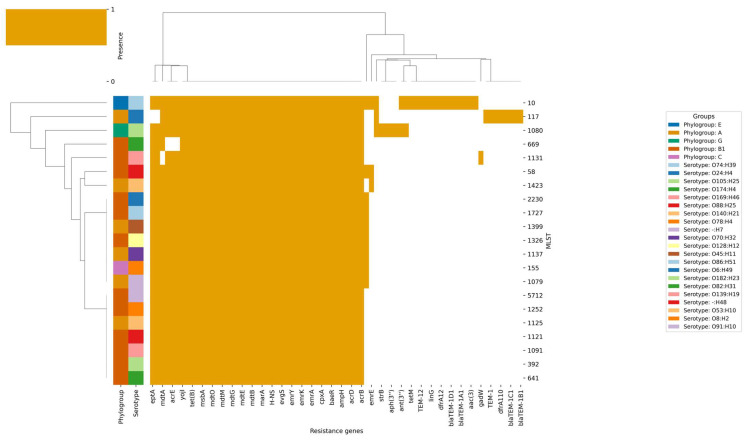
Heatmap and hierarchical clustering of resistance gene profiles in 46 *E. coli* isolates analyzed.

The second heatmap (Figure 7) illustrates the presence (ocher) of different resistance genes (mainly those encoding multidrug efflux pumps and their regulators, including *mdtA*, *mdtB*, *mdtC*, *mdtE*, *mdtF*, *mdtG*, *mdtM*, *mdtO*, *acrA*, *acrB*, *acrD*, *acrE*, *acrF*, and *marA*), across all isolates, irrespective of their origin, phylogroup, serotype, or MLST. The presence of these genes indicates that they are the core components of the *E. coli* genome, likely providing baseline resistance to a wide range of antibiotics, and are therefore evolutionarily conserved due to their essential role in bacterial survival across various environments. The heatmap illustrates a disparity in the distribution of acquired-resistance genes, including *tet(B)*, *aph(3’)*, *blaTEM-1*, *dfrA1*, and *aac(3)*, which are predominantly present in the mastitis associated strains. This suggests that the selective pressure from antibiotic treatment in mastitis likely facilitates the acquisition of these genes within specific clonal lineages. This observation is corroborated by the clustering patterns: isolates from mastitis cases, particularly those in phylogroups A and B1 and MLSTs such as ST1080 and ST117, tend to cluster together and are more prone to possess acquired-resistance genes. Conversely, isolates from healthy cows, including those with MLSTs ST1125, ST58, and ST392 and serotypes like O139:H19 and O8:H2, typically lack these genes and cluster separately, indicating their diminished exposure to antibiotics and reduced selective pressure for resistance acquisition. The serotype distribution underscores this trend, with mastitis-associated serotypes like O105:H25 and O78:H4 more commonly linked to acquired-resistance genes, whereas serotypes found in healthy cows, such as O139:H19 and O8:H2, are predominantly lacking these genes.

**Figure 8:**
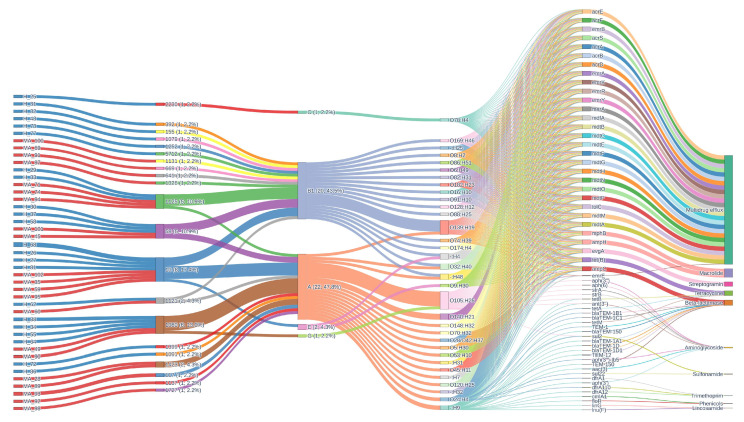
Alluvial diagram showing the distribution of resistance genes and their associations with phylogroups, sequence types, and resistance phenotypes.

The alluvial plot (Figure 8) provides a comprehensive analysis of resistance gene architecture, which reveals a highly intricate and dynamic landscape of antimicrobial resistance. This landscape is distinguished by a diversity of sequence types (STs), phylogroups, serotypes, and resistance mechanisms. ST1080 (17.4%), ST1080 (13.0%), ST58 (10.9%), ST1125 (10.9%), ST1121 (4.3%), and ST1423 (4.3%) are the most prevalent STs. A broad distribution of additional rare STs, each constituting 2.2% or less of the population, is also present. This diversity is reflected in the phylogroup composition, which is primarily composed of group A (47.8%) and B1 (43.5%), with minor contributions from E, C, and G. The complex genetic structure of the population is further emphasized by serotype analysis, which reveals the heterogeneity of the sampled isolates. O105:H25 (13.0%), O139:H19 (10.9%), O140:H21 (4.3%), and a variety of other serotypes, including non-typeable variants, all contribute to this structure.

The resistance gene landscape is primarily characterized by multidrug efflux systems, which are universally present in all isolates (100%). This is indicative of a strong selective pressure for broad-spectrum resistance and the potential impact of both clinical and environmental antimicrobial exposure. High frequencies of beta-lactamase, macrolide, streptogramin, and tetracycline resistance determinants, each of which is nearly ubiquitous within the dataset, complement the prevalence of multidrug efflux mechanisms. This indicates a significant burden of resistance, as the mean number of resistance mechanisms per sample is 5.2 and an average of 30.6 resistance genes per isolate, emphasizing the collection’s extensive genetic capacity for antimicrobial resistance. In contrast, sulfonamide, phenicol, trimethoprim, and lincosamide resistance mechanisms are sporadically distributed, suggesting either recent acquisition, limited selective pressure, or potential fitness costs associated with these mechanisms. Aminoglycoside resistance is less common, occurring in only 2.2–4.3% of isolates.

The genetic structure within the population is resolved through the comprehensive mapping of ST–phylogroup–serotype combinations. It is important to note that the co-occurrence of specific clonal backgrounds and serotypes with distinct resistance profiles is exemplified by combinations such as ST1080/A/O105:H25 (10.9%), ST1125/B1/O139:H19 (8.7%), and other prominent lineages. The flow of genetic information from STs through phylogroups and serotypes to specific resistance determinants is elegantly illustrated in the alluvial diagrams, which depict the architecture of resistance genes. This emphasizes the modularity and plasticity of the bacterial genome by illustrating the intricate relationship between functional gene content and clonal origin. Frequent horizontal gene transfer events and recombination are evident, resulting in a mosaic architecture of resistance determinants that can swiftly adapt to changing selective pressures. The figure also aids in the identification of potential locations for gene exchange and the emergence of multidrug-resistant clones, which have substantial implications for the management of antimicrobial resistance and public health. Beta-lactamase, macrolide, tetracycline, streptogramin, and multidrug efflux resistance are consistently present at 100% prevalence in both groups. However, phenicol and lincosamide resistance mechanisms are absent in MA_ but present at low frequency in H_.

### 3.4. Virulence Factors

**Figure 9:**
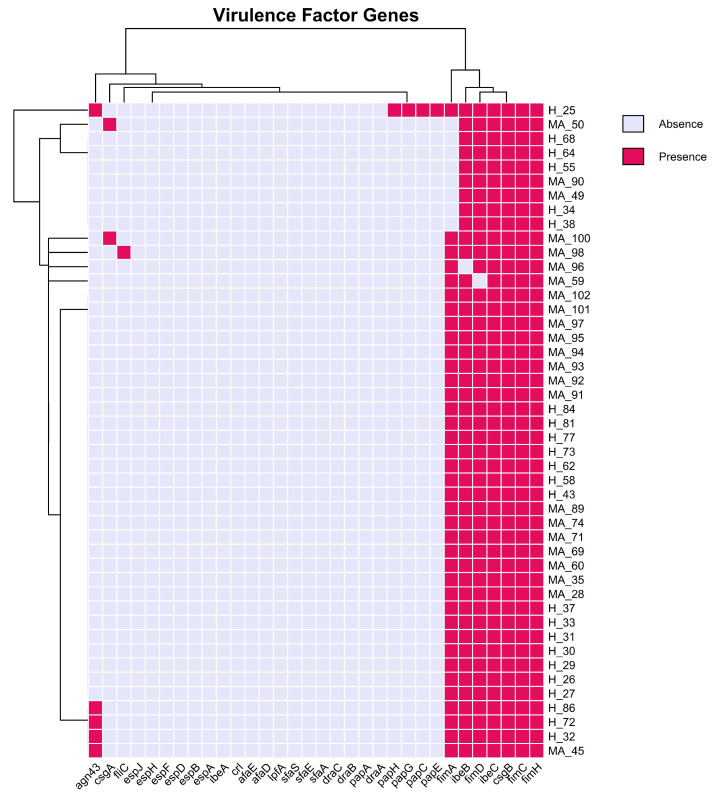
Heatmap of virulence factors in *E. coli*. Fuchsia, Presence; Liliac, Absence.

According to the literature [12,13,14], epithelial cell adhesion and invasion are critical factors for the onset and duration of mastitis in the mammary gland, but these features are not considered critical in the case of *E. coli* mastitis, being related mainly to LPS release. The results of our investigation on virulence adhesion factors (VFs) (Figure 9), such as the presence of fimbriae, flagella, adhesins, and type III secretion system (T3SS) confirm this information. Indeed, we could not identify a unique set of VFs that mastitis-associated *E. coli* strains carried, which could potentially account for the more pathogenic phenotype in comparison to healthy donors. Besides, it seems that no difference is present within strains, underlining the ubiquitous presence of type 1 fimbria for adhesion to epithelial cells (*fimACDH*), surface fibers for biofilm formation and adhesion enhancement (*csgB*), and VFs that facilitate invasion into the epithelial cells (*ibeB*, *ibeC*).

**Figure 10:**
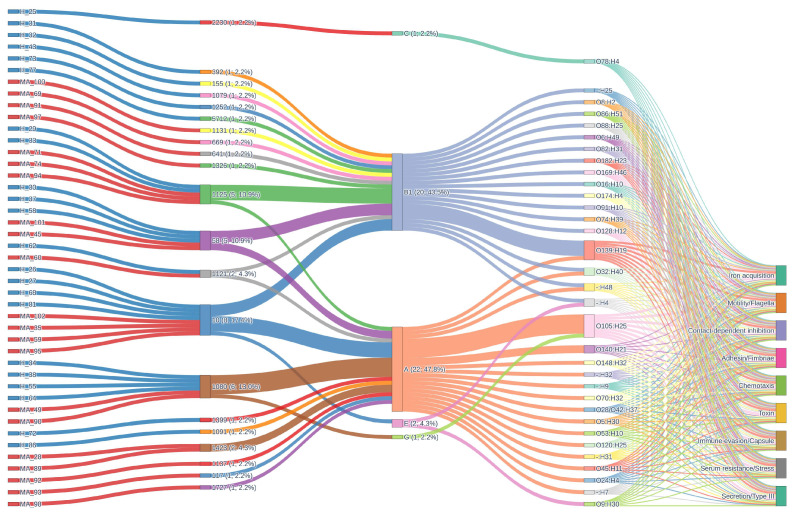
Alluvial diagram showing the distribution of virulence genes and their associations with phylogroups, sequence types, and resistance phenotypes.

The virulence gene distribution (Figure 10) is a landscape that is both highly conserved and not, with each isolate containing a diverse array of virulence determinants that collectively offer substantial pathogenic potential. The isolates considered possess an extensive genetic capacity for virulence, as evidenced by the mean number of virulence mechanisms per sample.

Genes associated with iron acquisition, motility/flagella, contact-dependent inhibition, adhesin/fimbriae, chemotaxis, toxin production, immune evasion/capsule formation, serum resistance/stress response, and type III secretion systems are included in this comprehensive repertoire. The conserved baseline of pathogenic potential that may be essential for survival, colonization, and infection in diverse host environments is suggested by the universal presence of these fundamental virulence mechanisms across all isolates, regardless of ST, phylogroup, or serotype. In addition to disclosing unique combinations and potential lineage-specific adaptations, the alluvial diagram for virulence genes vividly illustrates the convergence of diverse STs and serotypes onto a shared set of virulence mechanisms.

Across both the H_ and MA_ groups, the distribution of virulence mechanisms is remarkably consistent, with all main mechanisms present at a 100% prevalence.

Mapping ST–phylogroup–serotype combinations shows how the genetic structure of virulence varies across the population. ST1080/A/O105:H25 (10.9%) and ST1125/B1/O139:H19 (8.7%) are two examples of pairings that show that certain clonal backgrounds and serotypes can be found together with different virulence profiles. This method makes it possible to find epidemiologically relevant clones that might be connected to higher virulence, transmission, or the possibility of an outbreak.

The idea of a conserved core of virulence determinants is supported by the fact that different groups have several virulence mechanisms. All isolates have iron acquisition, motility/flagella, contact-dependent inhibition, adhesin/fimbriae, chemotaxis, toxin generation, immune evasion/capsule formation, serum resistance/stress response, and type III secretion systems.

## 4. Discussion

### 4.1. Phylogenetic Diversity and Core Genome Structure

To better understand the evolutionary context of the isolates, we first examined their phylogenetic distribution and core genome composition. Our comparative genomic study was done on a dataset of 46 *Escherichia coli* (*E. coli*) isolates from bovine milk in Italy. The dataset included an equal number of healthy (H_) and mastitis-affected animals (MA_). The key question our study tried to answer was whether mastitis-associated *E. coli* (MPEC) is a pathotype with unique genetic or virulence traits. This question is still very much up for dispute in the field, and our results can provide further data for this discussion. Our whole-genome sequencing and analysis showed that the technical quality was quite high. The genomes we got were almost complete and well-assembled, and the assembly statistics were the same for both health status groups. Most of the isolates clustered into phylogroups A and B1, which are the same two main groups that have been found in the literature for both commensal bovine and mastitis-associated strains. This supports the idea that there is a lot of phylogenetic diversity in these strains [8,15,16]. No unique clustering of MPEC based on phylogroup, MLST, or serotype was observed, echoing the consensus that MPEC are genetically heterogeneous and do not segregate into a monophyletic pathotype [8,17]. These results support previous findings that bovine *E. coli* populations are genetically diverse and that mastitis-associated isolates cannot be defined as a distinct evolutionary lineage.

### 4.2. Pan-Genome Composition and Accessory Gene Variability

Building on the phylogenetic results, we next explored the pan-genome composition to assess genomic plasticity and accessory gene variability. Our pan-genome analysis found 11,046 gene clusters, 2882 of which were core genes; this was in line with recent large-scale genome studies that showed how flexible *E. coli*’s genome is in cows [15]. Both healthy and mastitis-associated isolates had a lot of different accessory genes. These included cloud and shell genes that would have adaptive roles in the mammary niche. However, there was no collection of genes that were present in all MPEC and not in healthy cohorts. Many reports say that there is no conserved “mastitis virulence gene set”, and several comparative genomics studies have come to the same conclusion [16,18,19]. The virulence gene content of commensal and pathogenic isolates is very similar, and the known virulence determinants that are common in other ExPEC pathotypes (like Uropathogenic *E. coli* [UPEC] and Neonatal Meningitis-causing *E. coli* [NMEC]) are not often found or are not useful for distinguishing bovine mastitis [15,16,20,21]. Our accessory genome analysis, as well as recent publications, found enrichment in some strains for iron acquisition operons (*fec*, *iro*, *iuc*), adhesins, or capsule genes, but these features were distributed among both healthy and mastitis isolates [8,21]. This means considerable variation within the accessory genome, contributing to the persistent rise in the rarefaction curve and illustrating adaptation to diverse environments, acquisition of mobile genetic elements, and differing virulence factors.

### 4.3. Antimicrobial Resistance and Virulence Genes Patterns in Mastitis Pathogenicity

To investigate whether distinct antimicrobial resistance genes and virulence factors could explain mastitis pathogenicity, we examined gene patterns in comparison with existing ExPEC pathotypes.

One interesting result we found in our dataset, which is in line with what other researchers have found recently, is that all the bacteria carried antimicrobial resistance genes (AMRGs) and β-lactam resistance determinants (*ampC*, *ampH*). All the isolates had genes like *bacA* (resistance to bacitracin) and *pmrF* (resistance to polymyxin). This shows that milk-related *E. coli* has a lot of baseline antimicrobial resistance, but the number and variety of accessory resistance genes were not very different between healthy and MA animals. This is a pattern that has been seen in other research that looked at the resistance gene composition in bovine *E. coli* populations [20,21]. This pattern indicates that specific serotype-MLST-phylogroup combinations are more susceptible to acquiring and propagating resistance genes, maybe due to their ecological environment, genetic composition, and exposure to clinical interventions. The hierarchical clustering of genes and isolates on the heatmap (Figure 9) visually substantiates these associations, with core resistance genes forming a compact cluster present in all strains, while acquired-resistance genes exhibit smaller, more dispersed clusters linked to specific lineages and clinical contexts. Significantly, several healthy cow isolates group with mastitis-associated isolates, indicating either recent acquisition of resistance genes or possible misclassification, underscoring the dynamic nature of resistance gene spread. The biological findings are substantial: although intrinsic resistance mechanisms are universally present and likely crucial for fundamental bacterial physiology and survival, the intermittent yet epidemiologically significant occurrence of acquired-resistance genes in mastitis-associated isolates highlights the influence of antibiotic usage on the resistome of bovine *E. coli* populations. The elevated prevalence of acquired-resistance genes in mastitis cases has significant implications for animal health and antibiotic stewardship, indicating that existing treatment practices may unintentionally favor and disseminate resistant clones. The prevalence data in the dataset suggests that certain resistance mechanisms (multidrug efflux, beta-lactamase, macrolide, and tetracycline resistance) are universally or nearly universally distributed across both H_ and MA_ groups. This suggests that strong selective pressures and/or efficient horizontal gene transfer events are facilitating the dissemination of these determinants. Conversely, certain groups exhibit the absence or rarity of mechanisms such as phenicol and lincosamide resistance, which may be indicative of ecological or evolutionary constraints that restrict their dissemination. This pattern may indicate that environmental or host-associated factors significantly influence the resistance gene repertoire of bacterial populations, as it may be indicative of variations in selective pressures, antimicrobial use, or ecological context between the distinct groups.

Researchers are still very interested in finding specific genetic traits or phenotypes that are linked to some strains of *E. coli* being able to induce clinical mastitis. Some early investigations and reviews suggested that a new form of *E. coli* called “mammary pathogenic *E. coli*” (MPEC) might be emerging [6]. However, more recent and thorough genomic studies have not been able to consistently find unique virulence or fitness genes that are only found in mastitis isolates [8,15,21]. Our results strongly support this view; MA_ strains had only small increases in accessory genome size and gene count. This is consistent with the idea that sporadic acquisition of extra genetic material (e.g., plasmids, prophages, capsule islands) may change how pathogenic a strain is but does not define a pathotype.

### 4.4. Ecological and Epidemiological Insights

To interpret the genetic findings within a broader ecological and epidemiological perspective, we examined how environmental and host-related factors may shape the observed genomic patterns. Recent studies that looked at larger genome datasets have looked for accessory genes, single nucleotide polymorphisms, or gene expression profiles that could help tell MPEC apart. Researchers have suggested several genes that are interesting, such as the ferric dicitrate receptor operon (*fec*), genes that code for adhesins (*yad* fimbriae), or genes that are involved in making exopolysaccharides or capsules (e.g., *wbpl*, *capD*). These genes are mostly linked to disease isolates or the severity of clinical presentation [22]. In our study, we detected iron acquisition and adherence factors in several isolates, often more prevalent in MPEC, but these same genes also occurred occasionally in H_ strains: no single virulence determinant could predict phenotype. This emphasizes the bacterial genome’s adaptability and its ability to acquire virulence determinants through both vertical inheritance and horizontal acquisition. This implies that the selective pressures that influence the content of virulence genes are consistent across various ecological or host-associated contexts, or that the core set of virulence genes is necessary for fitness in a wide variety of environments. The presence of supplementary or accessory virulence determinants may alter pathogenesis in a strain-specific manner, resulting in variations in clinical outcomes, host range, or environmental persistence. A recent genome-wide study found that mutations and allelic variants of adhesins, biofilm genes (*pga* operon), and secretion system components were linked to either mild or severe mastitis in complicated ways [15,16]. However, these were affected by both phylogenetic background and accessory genome content, not by clear-cut pathotype-specific sequence markers [15]. Moreover, core genome phylogeny frequently exhibited a stronger correlation with variations in gene content than with disease association, reinforcing the notion that evolutionarily divergent lineages of *E. coli* may employ different genetic repertoires and tactics to prosper in the mammary gland [15,16]. Consequently, the emergence of a “pan-pathotype” with definitive markers seems improbable; instead, a mosaic and polygenic model of mastitis pathogenicity has gained substantial validation [15,16]. Our results align closely with genome-wide studies conducted by Leimbach et al. (2017) [8,15,16], Olson et al. (2024) [8,15,16], Kempf et al. (2016) [8,15,16], and others, which consistently did not identify MPEC as genetically separate from commensal bovine *E. coli*, highlighting the functional redundancy and adaptability of this species. The observed variability in surface antigens (O/H serotypes), sequence types, and accessory genes among mastitis isolates reinforces the conclusion that pathogenicity in the mammary gland arises not from the acquisition of a universal mastitis gene set, supporting the current knowledge that a non-genetic factor (LPS release) is the trigger for acute clinical mastitis. However, this does not exclude that a combination of core fitness genes, the capacity to evade host immune responses (e.g., serum resistance and biofilm formation), and the random acquisition of accessory modules may augment survival and pro-inflammatory potential [16,21,23].

### 4.5. Limitations and Future Perspectives

Although this study provides valuable genomic insights into the complexity of MPEC, we recognise that several limitations and open questions still remain. The scenario is significantly different from human ExPEC, EHEC, or EPEC pathotypes, where virulence gene signatures or toxins (e.g., *stx*, *eae*, *pap*) are distinctly associated with clinical syndromes. Our investigation, along with others, revealed that typical extraintestinal pathogenic *E. coli* (ExPEC) genes were frequently absent or shared between both groups, and no definitive “mastitis marker” was identified [24]. Traits such as the group III polysaccharide capsule observed in experimental hypervirulent MPEC models (e.g., M12 strain), while capable of providing increased fitness in infection models, are infrequently found in natural populations and do not constitute a population-wide mastitis-specific arsenal. Adding unusual and non-typeable variants to the population increases its genomic and functional diversity even more. These variants may act as reservoirs for new virulence genes or as steps in the evolution of new harmful lineages. As summarized in the alluvial plot of virulence genes, the mosaic structure of virulence determinants, reflect frequent recombination and horizontal gene transfer events. This shows how adaptable and modular the bacterial genome is. This plasticity makes it easier for organisms to quickly adjust to changes in their environment or host. It also allows hypervirulent or multidrug-resistant clones to appear, which have better fitness and disease-causing potential. The fact that these processes are found in so many places suggests that they are necessary for survival and success in a wide range of settings. Additional or accessory virulence genes can give organisms specialized capabilities that help them adapt to certain hosts or niches and improve the overall fitness and evolutionary success of the population.

Functional studies indicate that specific genes may influence the degree of mastitis (mild versus severe), and clusters related to adherence and exopolysaccharide synthesis have variable correlations with illness outcomes; nevertheless, these factors are neither exclusive nor essential for the hypothesized MPEC phenotype [15]. We considered isolates only from mild and severe mastitis, but not from acute mastitis, due to the need for very rapid treatment in this latter case. This may be considered a limitation since certain resistance and virulence genes are often associated with more severe clinical presentations.

Moreover, our data corroborate a robust alternative hypothesis suggesting that most of *E. coli* related to clinical mastitis are opportunistic commensals of the bovine gut or environment, adept at colonizing various niches. Therefore, other risk factors such as unproper milking practices and hygiene, physiological or pathological stress, in conjunction with stochastic host susceptibility, may lead to disease [8,16]. The absence of pathotype-defining features in MPEC is corroborated by evidence indicating that, apart from the predominance in phylogroups A and B1 (which also prevail in the H_ strains), neither horizontal gene transfer nor concentrated virulence gene distinguishes mastitis isolates, as supposed from existing literature [20,24].

## 5. Conclusions

In conclusion, our comparative genomic investigation of 46 *E. coli* isolates from healthy and mastitis dairy cows in Italy demonstrates significant genetic variety and flexibility within this crucial bovine-associated bacterial species. Despite methodologically sound study design and sampling, we identified no consistent genomic or phylogenetic markers capable of consistently differentiating mastitis-associated *E. coli* from those obtained from healthy animals. The pan-genome analysis uncovered a vast accessory genome and several cloud genes, alongside a restricted collection of universally shared core genes, further emphasizing the flexibility and mosaic nature of the *E. coli* population in the bovine mammary environment. Our study contributes to the consensus that the genetic basis of *E. coli* mastitis is intricate and multifaceted, with no singular genomic marker or virulence profile sufficiently accounting for disease association and underscores the dynamic evolutionary context *E. coli* encounters at the animal–environment interface in dairy systems.

## Figures and Tables

**Table 1:** Assembly statistics and genomic characteristics of 46 *Escherichia coli* isolates from *Bos taurus* milk, collected in Italy (2023–2024). For each isolate (23 from healthy: H_; 23 from mastitis-associated: MA_ animals), summary metrics are provided, including year of isolation, assembly level, assembly structure (Y: circular; N: linear), fragment number, genome size, GC content, CDS count, sequencing.

Isolate ID	BioSample	Genome	Yr of Isolation	Assembly Level	Circularity	No. of Fragments	Genome Size (Mbp)	GC (%)	CDS	COV ^a^ (x)	Com ^b^ (%)	Cont ^c^ (%)
H_EC25	SAMN47596209	JBPKIM000000000	2023	Contig	Y	2	4.98	51	4694	222	100	0.43
H_EC26	SAMN47596210	GCA_051912635.1	2023	Contig	Y	1	4.66	51	4319	149	100	0.31
H_EC27	SAMN47596211	JBPKIL000000000	2023	Contig	Y	3	5	51	4734	82	100	0.5
H_EC29	SAMN47596212	JBPKIK000000000	2023	Contig	N	2	4.94	51	4705	199	100	0.77
H_EC30	SAMN47596213	JBPKIJ000000000	2023	Contig	N	14	5.11	51	4829	79	100	0.18
H_EC31	SAMN47596214	JBPKII000000000	2023	Contig	N	2	5.07	51	4762	169	100	0.24
H_EC32	SAMN47596215	JBPKIH000000000	2023	Contig	N	2	4.89	51	4523	134	100	0.17
H_EC33	SAMN47596216	JBPKIG000000000	2023	Contig	N	6	5.02	51	4751	113	100	0.13
H_EC34	SAMN47596217	JBPKIF000000000	2023	Contig	N	17	5.06	51	4797	92	100	0.17
H_EC37	SAMN47596218	JBPKIE000000000	2023	Contig	Y	2	4.97	51	4663	116	100	0.14
H_EC38	SAMN47596219	JBPKID000000000	2023	Contig	Y	2	5.17	51	4976	176	100	0.18
MA_EC28	SAMN47596220	JBPKIC000000000	2023	Contig	N	5	5.09	51	4889	172	100	0.26
MA_EC35	SAMN47596221	JBPKIB000000000	2023	Contig	Y	2	5.13	51	4837	119	100	0.26
MA_EC45	SAMN47596222	JBPKIA000000000	2023	Contig	N	2	4.83	51	4519	167	100	0.37
MA_EC49	SAMN47596223	JBPKHZ000000000	2023	Contig	N	12	5.1	51	4915	119	100	0.21
MA_EC50	SAMN47596224	JBPKHY000000000	2023	Contig	N	15	5.16	51	4797	114	100	1.24
MA_EC59	SAMN47596225	JBPKHX000000000	2023	Contig	Y	2	5.07	51	4818	226	100	0.12
MA_EC60	SAMN47596226	JBPKHW000000000	2023	Contig	N	4	4.73	51	4467	163	100	0.08
MA_EC69	SAMN47596227	JBPKHV000000000	2023	Contig	N	8	4.85	51	4621	275	100	0.13
MA_EC71	SAMN47596228	JBPKHU000000000	2024	Contig	N	4	5.05	51	4823	126	100	0.34
MA_EC74	SAMN47596229	JBPKHT000000000	2024	Contig	N	3	4.83	51	4535	194	100	0.16
MA_EC89	SAMN47596230	JBPKHS000000000	2024	Scaffold	N	11	5.38	51	5192	201	100	0.5
MA_EC90	SAMN47596231	JBPKHR000000000	2024	Contig	N	3	5.09	51	4837	274	100	0.18
H_EC43	SAMN47596232	GCA_051912625.1	2023	Contig	Y	1	4.92	51	4600	318	100	0.15
H_EC55	SAMN47596233	JBPKHQ000000000	2023	Contig	N	45	5.23	52	4852	53	100	1.29
H_EC58	SAMN47596234	JBPKHP000000000	2023	Contig	N	3	4.77	51	4438	114	100	0.09
H_EC62	SAMN47596235	JBPKHO000000000	2023	Contig	N	9	5.1	51	4923	144	100	0.2
H_EC64	SAMN47596236	JBPKHN000000000	2023	Contig	N	6	4.92	51	4699	197	100	0.16
H_EC68	SAMN47596237	JBPKHM000000000	2023	Contig	Y	3	4.84	51	4531	130	100	0.03
H_EC72	SAMN47596238	JBPKHL000000000	2024	Contig	N	5	4.91	51	4576	243	100	0.21
H_EC73	SAMN47596239	JBPKHK000000000	2024	Contig	N	4	5.02	51	4718	145	100	0.25
H_EC77	SAMN47596240	GCA_051912615.1	2024	Scaffold	N	1	4.83	51	4475	233	100	0.1
H_EC81	SAMN47596241	JBPKHJ000000000	2024	Contig	N	4	4.69	51	4366	133	100	0
H_EC84	SAMN47596242	JBPKHI000000000	2024	Contig	N	2	5.02	51	4821	85	100	0.18
H_EC86	SAMN47596243	JBPKHH000000000	2024	Contig	Y	2	4.88	51	4632	394	100	0.21
MA_EC91	SAMN47596244	JBPKHG000000000	2024	Contig	N	2	4.78	51	4440	198	100	0.04
MA_EC92	SAMN47596245	JBPKHF000000000	2024	Contig	N	2	4.83	51	4562	271	100	0.12
MA_EC93	SAMN47596246	JBPKHE000000000	2024	Contig	Y	6	5.41	51	5121	297	100	0.48
MA_EC94	SAMN47596247	JBPKHD000000000	2024	Contig	N	3	5.01	51	4780	153	100	0.38
MA_EC95	SAMN47596248	JBPKHC000000000	2024	Contig	N	5	4.99	51	4740	284	100	0.12
MA_EC96	SAMN47596249	JBPKHB000000000	2024	Contig	N	5	4.6	51	4257	396	100	0.11
MA_EC97	SAMN47596250	JBPKHA000000000	2024	Contig	Y	3	4.85	51	4500	169	100	0.05
MA_EC98	SAMN47596251	JBPKGZ000000000	2024	Contig	N	5	4.83	51	4480	171	100	0.07
MA_EC100	SAMN47596252	JBPKGY000000000	2024	Contig	N	6	5.05	51	4592	167	100	0.18
MA_EC101	SAMN47596253	GCA_051912605.1	2024	Contig	N	1	4.79	51	4458	278	100	0.08
MA_EC102	SAMN47596254	GCA_051912595.1	2024	Contig	N	1	4.79	51	4504	237	100	0.22

Abbreviations: ^a^ coverage (COV), ^b^ completeness (Com), and ^c^ contamination (Cont).

## Data Availability

The whole-genome assemblies were deposited at GenBank repository under the bioproject PRJNA1242576 (https://www.ncbi.nlm.nih.gov/bioproject/PRJNA1242576, accessed on 16 September 2025).

## Supplementary Materials

The following supporting information can be downloaded at: https://www.mdpi.com/article/10.3390/pathogens14111181/s1, Figure S1: Rarefaction curve showing gene family accumulation with increasing numbers of H and MA genomes, highlighting an open pangenome and around 3196 gene families; Figure S2: U-shape frequency plot of gene family distribution.
