# Supplementary material for: Bovine Clinical E. coli Mastitis in Italian Dairy Herds Is Not Associated with a Specific Pathotype
Source: Pathogens. 2025 Nov 18;14(11):1181. doi: 10.3390/pathogens14111181 (PMC12655363; doi:10.3390/pathogens14111181)
Supplement: Supplementary file 1 [file pathogens-14-01181-s001.zip › Supplemental Figure S2.pdf]

Supplemental Figure S2: **U-shape frequency plot of gene family distribution.**

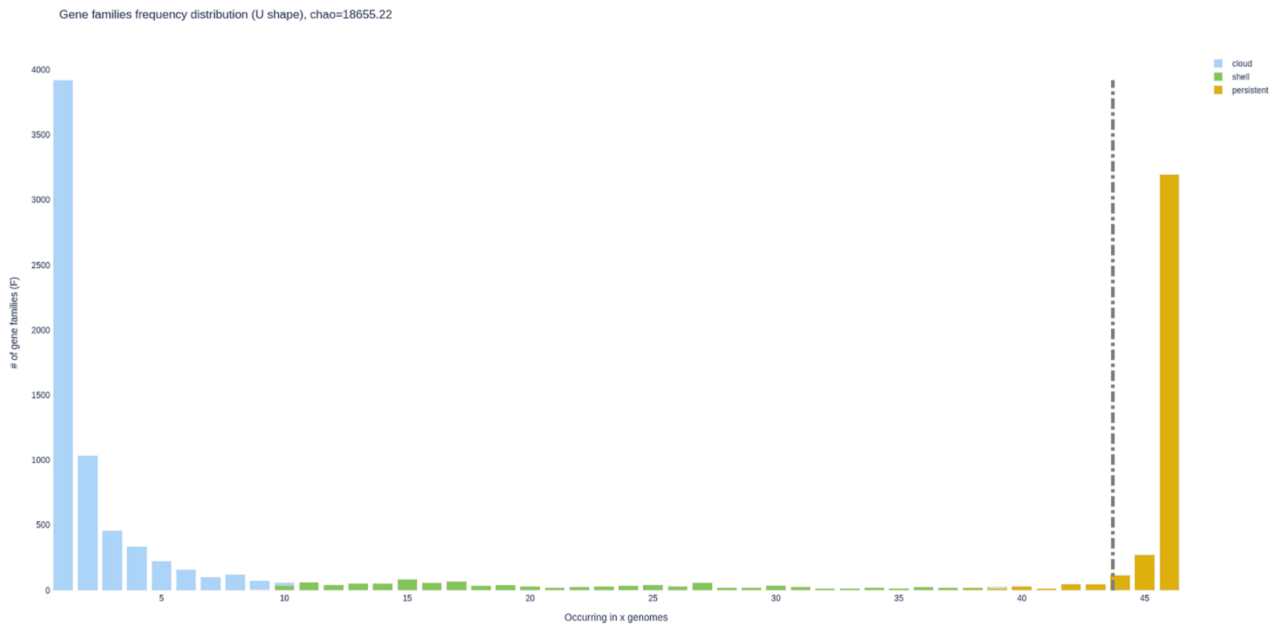

The x-axis shows how many genomes each gene family is in (1–46), whereas the y-axis counts families with that distribution. The blue bars on the left represent cloud gene families, found in a few genomes. The rightmost bar, steeply spiking in ocher at 46 genomes, represents persistent (core) genome, corresponding with baseline persistent family numbers (median: 3,739–3,849). The shell (green) between the cloud and persistent is scarce yet essential for population structuring. The largest class is cloud genome (approximately 4,000 gene families per genome) in the first bin (compared to 3,196 persistent/core families in all 46). The left-skew indicates significant gene influx, likely due to mobile elements, phages, and horizontal gene transfer, particularly in MA isolates with more rare families (median cloud families: 778 in MA_EC93, 134 in H_EC26).

Gene families from 11–37 strains together constitute the shell genome. The persistent genome (ocher bar) matches the rarefaction curve on the right; in fact, 3,196 families are detected in all strains.
