# Supplementary material for: Bovine Clinical E. coli Mastitis in Italian Dairy Herds Is Not Associated with a Specific Pathotype
Source: Pathogens. 2025 Nov 18;14(11):1181. doi: 10.3390/pathogens14111181 (PMC12655363; doi:10.3390/pathogens14111181)
Supplement: Supplementary file 1 [file pathogens-14-01181-s001.zip › Supplemental Figure S1.pdf]

Supplemental Figure S1: **Rarefaction curve showing gene family accumulation with increasing numbers of H and MA genomes, highlighting an open pangenome and around 3,196 gene families.**

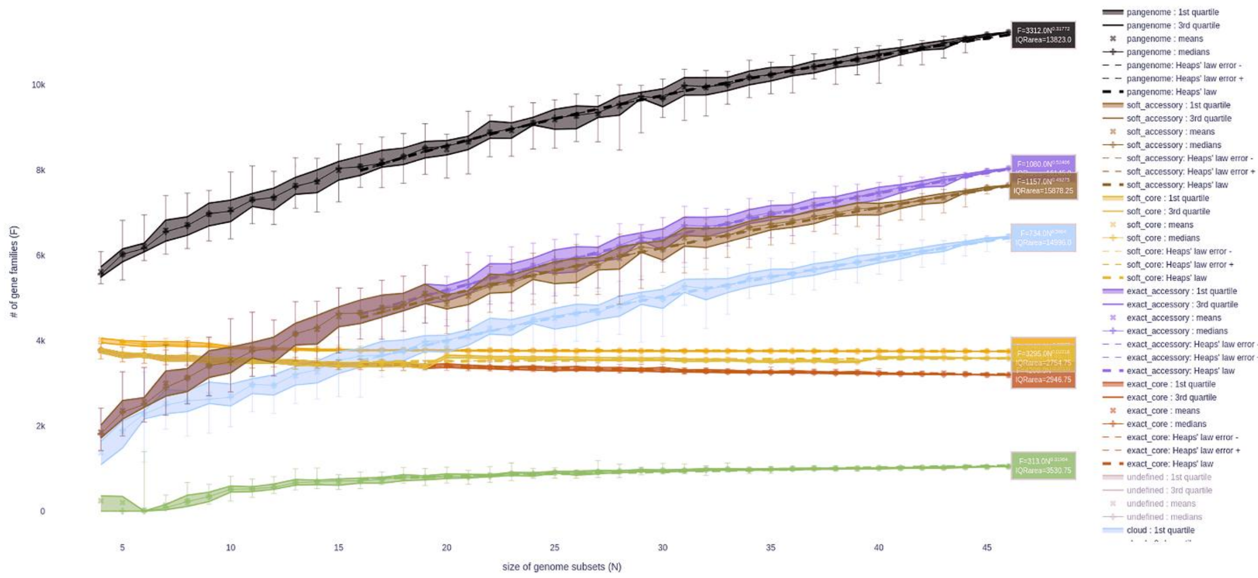

The black curve shows the whole pangenome, commencing at approximately 5,312 gene families and steadily increasing with the addition of more genomes, surpassing 10,000 families with 46 genomes included. The rarefaction trend, as indicated by the median, quartile, and Heaps' law estimations, has no plateau. This signifies an open pangenome: each new genome introduces unique gene families, highlighting substantial variety and continuous gene acquisition, in accordance with prior E. coli research. The soft accessory median attains about 4,100 gene families at maximum sampling, whereas soft core levels around 3,200 families. Both divisions expand with the inclusion of additional genomes, although the soft-core curve stabilizes sooner, in-dicating genes that are nearly ubiquitous across all genomes. The precise core (in green) swiftly attains a plateau at 3,196 gene families and remains constant. Approxi-mately 3,196 gene families are rigorously conserved across all 46 strains (both H and MA). These probably encompass genes critical for fundamental cellular functions. These trends are corroborated by genomic-level statistics. The median genome com-prises 4,500–4,800 genes, around 4,320–4,850 gene families and is classified within 3,196 precise core groups. Each genome generally comprises between 3,730 to 3,849 enduring families. MA_EC28 comprises 4,873 genes distributed among 4,712 families, including 3,572 soft core families and 3,822 persistent families. The entries of accessory and cloud gene families show huge variability, with the number of cloud families per genome ranging from a minimum of 134 to a maximum of 778 (e.g., H_EC64 vs. MA_EC93).
